# Gamma Delta T Cells (γδ T Cells) in Health and Disease: In Memory of Professor Wendy Havran

**DOI:** 10.3390/cells9122564

**Published:** 2020-11-30

**Authors:** Dieter Kabelitz

**Affiliations:** Institute of Immunology, Christian-Albrechts University of Kiel, D-24105 Kiel, Germany; dietrich.kabelitz@uksh.de; Tel.: +49-431-50031006

Gamma delta (γδ) T cells are a small subset of CD3-positive T cells in the peripheral blood but occur at increased frequency in mucosal tissues. The discovery of a second T-cell receptor 35 years ago was certainly unexpected [1,2,3,4]. Looking back, however, it is obvious that the scientific interest in γδ T-cell research has been undulating since then. A breakthrough of γδ T-cell research was the discovery that the major population of human γδ T cells does not recognize peptides presented by Human Leukocyte Antigen (HLA) molecules (like the conventional CD4^+^ or CD8^+^ T cells carrying the αβ T-cell receptor) but rather recognize nonpeptidic phosphorylated molecules secreted by bacteria [5]. Such “phosphoantigens” have been discovered not only in many bacteria and some parasites but also in eukaryotic cells as intermediates of the mevalonate pathway of cholesterol synthesis [6]. Overproduction of phosphoantigens in tumor cells due to a dysregulated mevalonate pathway has been shown to activate tumor-reactive γδ T cells [7]. How such phosphoantigens are recognized by γδ T cells has been a mystery for many years. A landmark paper by Harly and colleagues identified an indispensable role of the butyrophilin family transmembrane molecules, specifically butyrophilin (BTN) 3A1 [8]. Exciting new studies have recently highlighted a similarly indispensable role of another BTN member, BTN2A1 [9,10]. Other studies have shown that BTN-like molecules are also very important for the regulation of tissue-resident human γδ T cells [11]. Taken together, there has been tremendous recent progress in the elucidation of the molecular pathways of how γδ T cells (as opposed to conventional αβ T cells) are activated and influenced by the local micromilieu. From a translational perspective, the potential advantage of γδ T cells for (cancer) immunotherapy has long been recognized. The more detailed knowledge of how γδ T cells recognize transformed cells independently of HLA restriction has certainly fueled this interest, as reflected by the increasing number of companies devoted to clinical exploration of γδ T-cell immunotherapy [12]. 

This Special Issue of *Cells* comprises 17 original papers and review articles on various aspects of γδ T cells in health and disease. In view of the recent and exciting progress in the field, I thought that this would be a wonderful time to collect contributions from leading experts to share their results and amazing new insights with the scientific community. While preparing this Special Issue, we had to mourn the sudden death of Wendy Havran, a giant in the field of γδ T-cell research and an exceptional and close friend to many of us in the γδ T-cell world. I appreciate the support of the publishers to devote this Special Issue to the memory of Professor Wendy Havran.

The first paper is an appraisal of Professor Wendy Havran, written by her long-term associate Deborah Witherden. This short editorial illustrates the extraordinary personality of Wendy Havran both as a scientist and as a mentor for a large number of trainees in her lab, many of whom have moved on to take leadership positions at universities or in industry [13].

Four reviews and two original papers in this Special Issue concentrate on characteristics of γδ T cells under physiologic conditions, and how γδ T cells are activated and interact with other immune cells. Fonseca and colleagues present a thorough phenotypic analysis of γδ T cells in a population of 30 Caucasian blood donors with a mean age of 47 years. Even though this is a relatively small cohort, the results clearly indicate the broad range of variability of γδ T-cell subset representation (e.g., Vδ1 versus Vδ2) in healthy adult blood donors. This paper also summarizes the available literature about parameters possibly influencing immune cell composition including γδ T cells, such as age, gender, and ethnicity [14]. A puzzling issue in γδ T-cell research is the analysis of the T-cell receptor (TCR) repertoire under physiological and pathological conditions. As compared to the conventional αβ T cells, the number of expressed Vγ and Vδ genes is very small; there are only six Vγ genes in humans which can be expressed at the protein level, and monoclonal antibodies have been generated to detect the complete expressed human Vγ repertoire [15]. This notwithstanding, it is obvious that the recent introduction of high-throughput TCR sequencing methodology has tremendously advanced our knowledge of the clonal composition of the γδ TCR repertoire and how it changes during development and aging as well as in pathological situations like infection or tumorigenesis. The current state of the art of this exciting topic is summarized in the expert review by Fichtner et al. [16]. The representation of γδ T-cell subsets and utilization of the γδ TCR are known to be influenced by age. In humans, there are well-characterized alterations in the relative proportion of major γδ T-cell subsets Vδ1 and Vδ2 not only at different stages of gestation but also postnatally during the transition from childhood to adulthood and old age. While the underlying mechanisms of age-dependent alterations in the γδ T-cell compartment are not precisely known, exposure to chronic infections like cytomegalovirus (CMV) is likely to play a role, as discussed by Xu et al. in this Special Issue [17].

How do γδ T cells interact with other immune cells? T cells in general are important for providing helper signals for the activation of antibody-producing B cells. It is well known that γδ T cells can interact with B cells through multiple costimulatory pathways including CD40/CD40-ligand, inducible T-cell costimulatory (ICOS/ICOS-ligand), or CD86/CD28. Together with cytokines produced by γδ T cells, this may drive the differentiation of B cells into antibody-secreting plasma cells. The interaction is reciprocal, however, and B cells may thus also modulate γδ T-cell activation. This as well as interesting additional aspects, namely the possible role of γδ T cells in the regulation of autoantibody responses in human autoimmune diseases, are discussed in the paper by Rampoldi et al. [18].

As mentioned above, human Vγ9Vδ2 T cells specifically recognize microbial or tumor-derived phosphoantigens. Members of the butyrophilin transmembrane molecules play an indispensable role in this process. The molecular knowledge of how this is accomplished has tremendously grown in recent years. We now know that it is not only the BTN3A1 molecule that is important but rather that BTN2A1 is equally important. An update of the molecular basis of phosphoantigen recognition by Vγ9Vδ2 T cells is provided by Herrmann et al [19]. These new insights also have practical implications when it comes to the intentional activation (or inhibition) of γδ T cells by therapeutic manipulation. TCR-dependent recognition of phosphoantigens is key to the selective activation of Vγ9Vδ2 T cells. However, activation can be modulated by costimulatory signals. In this respect, Serrano and coworkers have investigated the costimulatory effect of ligands for selected Toll-like receptors (TLRs), notably TLR8. They observed that such TLR8 ligands rapidly induced interferon-γ production in γδ T-cells within the total population of peripheral blood mononuclear cells and also costimulated the phosphoantigen-induced interferon-γ production. Quite strikingly, the same TLR8 ligands inhibited the proliferative expansion of γδ T cells in vitro. The detailed analysis revealed a critical role of monocytes in both situations, as reported in the paper by Serrano et al. [20].

Although Vγ9Vδ2 T cells recognizing phosphoantigens in the context of butyrophilin molecules are the dominant γδ T-cell subset in human peripheral blood, it is well known that major populations of γδ T cells are localized in tissues, both in mice and humans. Here, γδ T cells seem to exert local immunosurveillance by constantly monitoring tissue integrity. An impressive example is the dendritic epidermal T cells (DETCs) localized in the epidermis of mice, which basically represent a dense network of cells morphologically resembling dendritic cells. In fact, however, DETCs represent a clonal population of γδ T cells expressing a monomorphic γδ TCR. Professor Wendy Havran has pioneered the discovery and functional analysis of murine DETCs [21], and her group was the first to identify self-ligands that are recognized by the DETCs [22]. Over the years, Wendy has substantially contributed to the molecular characterization of ligands involved in the activation of DETCs, as well as contributing to the identification of what role these cells play in tissue repair and wound healing [23]. The paper by Wendy Havran’s associates Margarete Johnson and Deborah Witherden provides an insightful review on the significance of tissue-resident γδ T cells in the epidermis, intestinal epithelium, and adipose tissue [24]. Obviously, however, phosphoantigen-reactive human γδ T cells also mediate stress surveillance, since sufficiently high levels of endogenous phosphoantigens (mainly isopentenyl pyrophosphate (IPP)) to activate γδ T cells are only produced by stressed and transformed cells, not by healthy cells. Another component of the stress surveillance is the recognition of stress-inducible ligands for the NKG2D receptor expressed on most Vγ9Vδ2 T cells. NKG2D ligands like major histocompatibility complex (MHC) class I-related chain A and B (MICA/B) are expressed upon stress or cellular transformation, thereby enabling NKG2D-positive γδ T cells to recognize and respond to stressed/transformed cells. The implications of the various recognition systems for the stress surveillance function of human Vγ9Vδ2 γδ T cells are discussed by Nussbaumer and Thurnher [25].

The second part of this Special Issue comprises nine review articles and original papers on the role of γδ T cells in disease. The majority of these articles address the role of γδ T cells in cancer immunity and how these cells can be exploited for immunotherapy. Obviously, however, γδ T cells are also involved in other diseases, notably autoimmune diseases and viral infections. It was already mentioned that γδ T cells might have a dedicated role in the production of autoantibodies [18]. In continuation, the review by Ilan Bank highlights the multifaceted role of γδ T cells in various autoimmune diseases, including rheumatoid arthritis, ankylosing spondylitis, and systemic lupus erythematosus. In many instances, it is unclear whether alterations in the γδ T-cell compartment are a contributing factor in pathophysiology or are secondary to alterations of other immune cell parameters. This review provides an in-depth overview of what is currently known about γδ T cells in autoimmune diseases, both in experimental model systems as well as in patients [26]. Alterations in the γδ T-cell compartment are observed during viral infections, notably in HIV-infected people. It has been known for some time that there is a reduction in the proportion of Vδ2 T cells in HIV-infected donors, frequently associated with a relative and also absolute increase in the proportion of Vδ1 T cells. Given that Vδ2 T cells contribute to anti-microbial immunity, it is argued that the depletion of Vδ2 T cells reflects the exposure to multiple microbes including apathogenic mycobacteria, leading to chronic activation and exhaustion of Vδ2 T cells. Importantly, the paper by Clohosey *et al*. provides interesting insights how effective anti-retrovial therapy also reconstitutes the functional activity of γδ T cells [27]. 

Given the above-discussed role of γδ T cells in immunosurveillance, it comes as no surprise that tremendous efforts have been devoted to delineating the role of γδ T cells in tumorigenesis and developing strategies for their potential use in cancer immunotherapy. One of the first studies to clearly show that γδ T cells are crucial in local stress surveillance was the demonstration that mice lacking γδ T cells (including DETCs) are highly susceptible to multiple regimens of skin carcinogenesis [28]. When addressing the potential role of γδ T cells in the immune response to cancer, an important aspect is the analysis of γδ T cells within the tumor microenvironment in situ. Some years ago, a highly cited paper described the abundance of tumor-associated γδ T cells at the transcriptomic level as the single most favorable prognostic marker out of 22 distinct leukocyte subsets in 18,000 tumor samples across 39 different cancer types [29]. Subsequent studies, however, uncovered methodological limitations of this study and—using more detailed and refined computational approaches—described a more selected beneficial role of γδ T cells in certain cancer types [30]. While the transcriptome analysis of immune cell abundance is certainly a powerful tool, it seems equally important to detect γδ T cells and to analyze their spatial localization within different regions in the tumor by immunohistology and more sophisticated technologies such as high-content imaging and quantitative whole-slide imaging analysis [31]. In this respect, the paper by Chabab et al. is a very important contribution to the Special Issue. They have optimized the detection of γδ T cells by immunohistochemistry and used this approach to quantify the numbers of γδ T cells in healthy tissue and in breast, colorectal, ovarian, and pancreatic cancer. Their interesting results underscore the role of different tumor environments in the recruitment of γδ T cells into the tumor [32]. 

It is well known that the tumor microenvironment contains multiple elements that are directed towards suppressing efficient antitumor immune responses. These include tumor-associated macrophages (TAM) and myeloid-derived suppressor cells (MDSC) which exert their suppressive effects through multiple pathways, including the production of inhibitory cytokines like transforming growth factor-β [33]. Suppressive mechanisms mediated by the tumor cells themselves or tumor-associated stroma can also negatively impact the antitumor activity of γδ T cells [34]. Jonescheit and colleagues have investigated the role of indoleamine-2,3-dioxygenase (IDO) and its metabolite kynurenine on the cytotoxic effector activity of human Vγ9Vδ2 T cells against pancreatic adenocarcinoma cells. Their results indicate variable expression of IDO in tumor cells and an inhibitory effect of kynurenine on γδ T-cell cytotoxicity, thereby raising the possibility that IDO expression might contribute to tumor escape from γδ T-cell attack [35].

While it is important to understand tumor immune escape mechanisms, it seems equally important to increase the activation and effector functions of γδ T cells. In this respect, our group has recently reported beneficial effects of vitamin C, which acts as both antioxidant and epigenetic modifier [36]. Ma and colleagues have investigated the effects of mistletoe extracts on the activation of human Vγ9Vδ2 T cells. Mistletoe extracts are popular adjuvants in cancer therapy in certain countries, including Germany. In fact, we reported in 1996 that heat-treated mistletoe extracts contain phosphatase-sensitive and proteinase-resistant substances which activate the same subset of human γδ T cells that is known to recognize microbial or tumor-derived phosphoantigens in a BTN3A-dependent manner [37]. In their study, Ma et al. used refined and state-of-the-art approaches to characterize in detail the Vγ9Vδ2 T-cell response to mistletoe extracts, supporting the idea that such herbal drugs might help to boost the antitumor response of γδ T cells [38].

Three additional review articles in this Special Issue highlight the current knowledge on the role of γδ T-cells in tumor immunity and the current concepts of how to bring γδ T cells into clinical application for the treatment of cancer patients [39,40,41]. Importantly, mice do not have γδ T cells which respond to the phosphoantigens known to be the most potent and selective ligands for the human Vγ9Vδ2 T cells. Therefore, conventional mouse models cannot be used to address the role of human Vγ9Vδ2 T cells in antitumor immunity. As an alternative, researchers have used various immunodeficient mouse strains for xenografting human tumor cells and human Vγ9Vδ2 T cells. The review by Künkele et al. also provides a detailed overview of various xenograft models that have been used to study the antitumor reactivity of human γδ T cells [39]. These reviews also contain detailed overviews of the current status of clinical trials with in vitro expanded γδ T cells (adoptive transfer) or in vivo activation of γδ T cells with aminobisphosphonates or phosphoantigens, and they discuss novel approaches to possibly increase the efficacy of γδ T-cell-based immunotherapy [39,40,41]. To date, the clinical benefit of cancer immunotherapy with γδ T cells is very limited, and there is certainly room for improvement. However, there are multiple strategies on the horizon which might help to translate γδ T-cell immunotherapy into a clinical success, which is also reflected by the increasing number of companies focusing on γδ T-cell immunotherapy [12]. The HLA independence is considered a decisive advantage of γδ T cells, as adoptive transfer of γδ T cells from healthy donors to cancer patients across HLA barriers can be considered and, in fact, has already been performed [42]. 

The collection of original papers and review articles in this Special Issue provide an up-to-date overview of “hot topics” in current γδ T-cell research. Not surprisingly, several contributions focus on the role of γδ T cells in immunosurveillance and cancer immunity. The tumor-derived ligands recognized by γδ T cells and their HLA independence qualify γδ T cells as immune cells important for tumor control and distinct from classical αβ T cells and NK cells. We are facing exciting developments in the field of cancer immunotherapy, and the recent foundation of several companies focusing on the antitumor activity of γδ T cells firmly supports the notion that γδ T cells will play an important role in cell-based immunotherapy.
